# Traditional Chinese medicine in osteoporosis: from pathogenesis to potential activity

**DOI:** 10.3389/fphar.2024.1370900

**Published:** 2024-04-02

**Authors:** Gang Cao, ShaoQi Hu, Yan Ning, Xinyue Dou, Chuan Ding, Lu Wang, Zeping Wang, Xianan Sang, Qiao Yang, Jiangnan Shi, Min Hao, Xin Han

**Affiliations:** ^1^ Innovative Institute of Chinese Medicine and Pharmacy, Chengdu University of Traditional Chinese Medicine, Chengdu, China; ^2^ School of Pharmacy, Zhejiang Chinese Medical University, Hangzhou, China

**Keywords:** osteoporosis, pathogenesis, pathogenic factors, traditional Chinese medicine, treatment

## Abstract

Osteoporosis characterized by decreased bone density and mass, is a systemic bone disease with the destruction of microstructure and increase in fragility. Osteoporosis is attributed to multiple causes, including aging, inflammation, diabetes mellitus, and other factors induced by the adverse effects of medications. Without treatment, osteoporosis will further progress and bring great trouble to human life. Due to the various causes, the treatment of osteoporosis is mainly aimed at improving bone metabolism, inhibiting bone resorption, and promoting bone formation. Although the currently approved drugs can reduce the risk of fragility fractures in individuals, a single drug has limitations in terms of safety and effectiveness. By contrast, traditional Chinese medicine (TCM), a characteristic discipline in China, including syndrome differentiation, Chinese medicine prescription, and active ingredients, shows unique advantages in the treatment of osteoporosis and has received attention all over the world. Therefore, this review summarized the pathogenic factors, pathogenesis, therapy limitations, and advantages of TCM, aiming at providing new ideas for the prevention and treatment of OP.

## Graphical Abstract

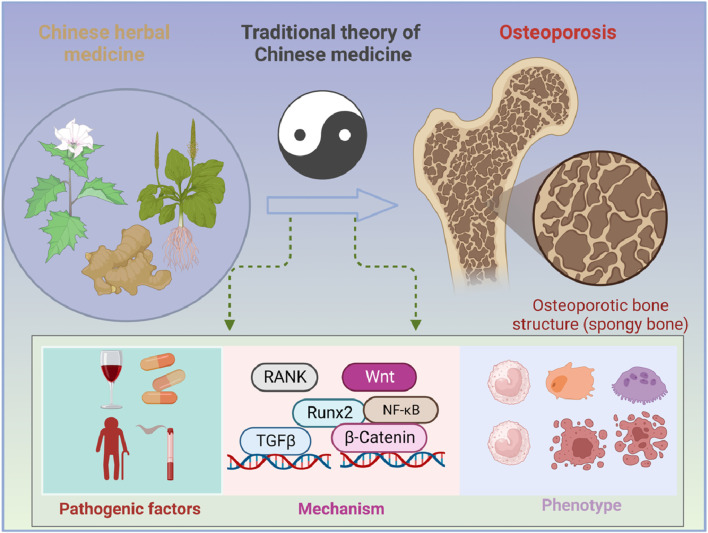

## 1 Introduction

Osteoporosis (OP), characterized by the decrease in bone mineral density (BMD) and disorders of the bone microstructure, is a chronic metabolic bone disease (Noh et al., 2020). OP is a result of the imbalance of bone homeostasis that is maintained by coordinated cycles of bone resorption and formation and can contribute to the increase in bone fragility as well as the risk of fractures (El-Gazzar and Högler, 2021; Zhang et al., 2023). These fragility fractures lead to a disproportionately high mortality rate and drastically reduce the quality of life, all of which make osteoporosis a growing and enormous threat to public health that affects 200 million people worldwide (Muñoz et al., 2020). From the perspective of etiology, osteoporosis can be divided into two types: primary and secondary (Eastell et al., 2016; Compston et al., 2019). Among them, primary osteoporosis is a result of aging, and its most common form is postmenopausal osteoporosis (PMOP), which is caused by the decrease in estrogen secretion after menopause (Compston et al., 2019), while secondary osteoporosis occurs when BMD is reduced by other factors such as drugs (Compston et al., 2019; S., 2011). Based on various induction factors of osteoporosis, a series of therapeutic drugs for osteoporosis have been developed for clinical practice, including estrogen, calcitonin, bisphosphonates, and teriparatide (Khosla and Hofbauer, 2017). Among these, estrogens, used in the prevention and therapy of osteoporosis, have certain side effects, including increased risk of cardiovascular events and breast cancer (Rossouw et al., 2002). Calcitonin was developed based on animal and human studies and is now rarely used for the treatment of osteoporosis due to its limited efficacy on osteoporosis and concerns that its long-term use may increase the risk of cancer (Chesnut et al., 2000). In addition, bisphosphonates are the most widely used drugs, but poor adherence is a major limiting factor for their treatment of osteoporosis, which is largely associated with gastrointestinal adverse events (Cramer et al., 2007). Moreover, treatment with high doses of teriparatide may increase the risk of developing osteosarcoma in growing rodents (Vahle et al., 2016). Therefore, it is urgent to seek some effective drugs with few side effects for the treatment of osteoporosis. Despite some progress in the treatment of osteoporosis, the side effects of these drugs are of concern (Khosla and Hofbauer, 2017). Therefore, exploring the pathogenesis of OP and finding its therapeutic drugs are crucial.

OP is closely associated with aging, endocrine diseases, chronic kidney diseases, gastrointestinal diseases, and so on (Lane, 2006). It is worth mentioning that osteoporosis is often accompanied by pain, spinal deformity, and fracture, even limitation of movement and disability, all of which seriously affect the quality of daily life (Tsai et al., 2019). Under the inducement of these factors, the formation of OP is associated with osteoblasts that maintain functional cells of bone formation and osteoclasts that participate in bone resorption. Osteoblasts are the main functional cells in bone for bone resorption. Osteoblasts play an important role in the synthesis, secretion, and mineralization of the bone matrix, while osteoclasts, known as bone-resorbing cells, are a component of bone tissue and mainly regulate the function of bone resorption (Chotiyarnwong and McCloskey, 2020). In OP, osteoclasts accomplish the transformation from bone resorption to bone formation by transmitting coupling signals to osteoblasts (Ikebuchi et al., 2018). During this process, the receptor activator of NF-κB (RANK)/receptor activator of the NF-κB ligand (RANKL)/osteoprotegerin (OPG) axis plays a key role (Zhao et al., 2020). Studies showed that the inhibition of RANKL can be a therapeutic strategy for excessive bone resorption, such as recombinant, which is still in the research stage due to its uncertain side effects. Therefore, it is urgent to seek some effective drugs with few side effects for the treatment of OP (He et al., 2019).

Traditional Chinese medicine (TCM), with few side effects, has unique advantages in the treatment of chronic disease. Studies showed that TCM has a long history in the prevention and treatment of OP (Zhang et al., 2016), such as *Eucommiae Folium*, *Cornus officinalis*, and *Radix Angelicae sinensis*. Moreover, TCM treatment based on syndrome differentiation is the accumulation of clinical practice gathered over centuries (Fu et al., 2021) and has specific advantages in the treatment of OP. In this review, we summarized the pathogenic factors, pathogenesis, therapy limitations, and advantages of TCM, aiming at providing new ideas for the prevention and treatment of OP.

## 2 Pathogenic factors of osteoporosis

### 2.1 Aging is a major cause of osteoporosis

The skeletal system grows rapidly, mainly from the postnatal period to puberty after birth and adolescence, reaches its peak at about 35 years of age (Wayne Sampson, 2002), and then, gradually decreases with age in both men and women (Yamakawa et al., 2020). Therefore, aging is a baseline risk factor in the development of OP and bone fracture, as well as a predictor of poor outcomes after fracture (VanderWalde and Hurria, 2011). In women, primary OP, also called postmenopausal OP, is mainly induced by menopause plus the cessation of ovarian function, specifically, a decline in postmenopausal ovarian endocrine function, resulting in the decrease in Es level and leading to greater bone resorption than bone formation (Wu et al., 2021). Of note, up to one-third of fragility fractures occur in older men and are usually accompanied by severe osteoporotic fractures, especially hip fractures. Due to these problems, OP in the elderly is an important threat to the life quality of individual patients and a huge burden to society (Kaufman, 2021). Therefore, exploring the pathogenesis of aging-related OP is crucial for the treatment of OP. Qadir et al. stated that due to aging, bone marrow stromal cells were more likely to differentiate into adipocytes rather than osteoblasts, which contributes to the decrease in bone formation, leading to the development of senile OP (Qadir et al., 2020). Moreover, with aging, the excessive accumulation of reactive oxygen species (ROS), interleukin-6 (IL-6), tumor necrosis factor-α (TNF-α), and other cytokines in cells will affect the differentiation of osteoclasts and the formation of osteoblasts, leading to osteoporotic bone loss (Yu and Wang, 2016). Therefore, inflammation is another cause of osteoporosis.

### 2.2 Inflammation is involved in the development of osteoporosis

Innate immune cells are the major source of proinflammatory factors, such as IL-6 and TNF-α, and can immediately respond to various challenges in the body, which has been considered one of the main inducements of skeletal diseases (Hato and Dagher, 2015). In OP, osteoblasts undergo programmed necrosis and release NOD-like receptor protein 3 (NLRP3), thereby resulting in inflammatory responses. During these processes, IL-1β and IL-18 were cleaved by caspase-1 and turned into mature forms that could be released into the extracellular environment, which promoted excessive bone resorption (Vijayaraj et al., 2021). Moreover, other immune cells, such as DCs, macrophages, and monocytes, can also take part in osteoclast formation because they share a common developmental niche (Ponzetti and Rucci, 2019). Analogous eosinophils, mast cells, and neutrophils could also contribute to the development of OP (Yu et al., 2015; Ragipoglu et al., 2020). It can be seen that inflammation plays a critical role in OP due to its role in bone loss and osteoblast function (Amarasekara et al., 2015), which is characterized by BMD reduction and the production of cytokines in diseases such as periodontitis (Yu and Wang, 2022) and rheumatoid arthritis (RA) (Forsblad D'Elia et al., 2003).

### 2.3 OP is often a complication of diabetes mellitus

OP is also closely associated with diabetes mellitus (DM), which is characterized by polyphagia, polyuria, and hyperglycemia (Fang et al., 2021). DM is usually divided into two types: type 1 diabetes mellitus (T1DM) and type 2 diabetes. Among them, T1DM is known as insulin-dependent diabetes and can induce bone loss due to calcium and phosphorus imbalance (Lecka-Czernik, 2017), while type 2 diabetes is non-insulin-dependent diabetes mellitus that is considered a risk factor for OP (Barrett-Connor E, 1992), mainly due to that increased obesity in diabetic patients will affect the function of osteoblasts and osteoclasts (Rathinavelu et al., 2018). In addition, a variety of chronic complications related to diabetes mellitus, such as diabetic liver disease (fatty liver), diabetic nephropathy (Paschou et al., 2017), microvascular disease (Samakkarnthai et al., 2020), and diabetic neuropathy (Paschou et al., 2017), can also contribute to the development of OP.

### 2.4 Other factors contribute to osteoporosis

**FIGURE 1:**
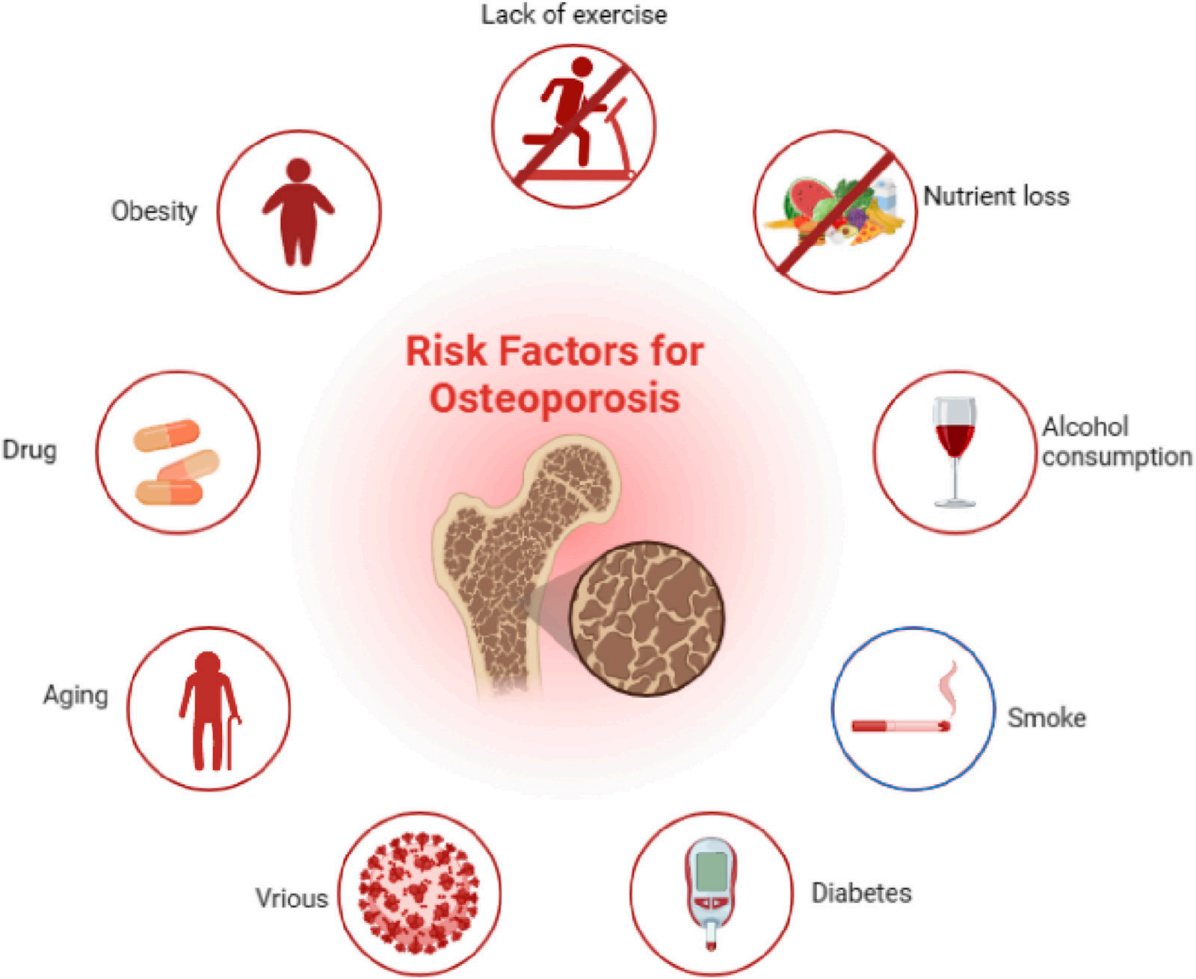
Pathogenic factors of osteoporosis, such as aging, inflammation, and diabetes mellitus, could contribute to the development of osteoporosis.

In addition, other factors, such as cerebral apoplexy, breast cancer (Sozel and Yilmaz, 2021), decompensated cirrhosis (Yang and Kim, 2021), gastrointestinal disease (Klaus et al., 2002), bowel disease, celiac disease, and hyperuricemia (Lee et al., 2021) and its induction divisors (smoking, alcohol, virus, etc.) (Lo et al., 2020), can also contribute to various degrees of osteoporosis. It is worth mentioning that the current treatment drugs for the above diseases include rosiglitazone, bisphosphonate (Sheu et al., 2022), unfractionated heparin, and low-molecular-weight heparin (Zhang B. et al., 2021). Proton pump inhibitors also play critical roles in the development of OP. In addition, a study showed that weight was also closely associated with the incidence of OP (Andreoli et al., 2011), which is due to that a high body mass index is related to high BMD and reduction of fracture risk in postmenopausal females (Tariq et al., 2017). Furthermore, other factors that affect body weight are also involved in the development of OP, such as nutrition and exercise. To be specific, malnutrition caused by low-protein diets can reduce the expression of insulin-like growth factor 1 (IGF-1), suppress the absorption of intestinal calcium and phosphorus, and inhibit bone calcification (Muñoz and Argente, 2002); exercise can regulate the biological activity of osteoblasts, increase mass accumulation of bone, and prolong bone turnover rate; furthermore, long-term plus regular weight-bearing exercise can increase BMD and reduce bone loss (Stein and Shane, 2003) (Figure 1).

## 3 Treatment of OP

Due to the variety of pathogenic factors of OPG, the application of TCM and Western medicine in the treatment of OP is also very different. For Western medicine, the treatment strategy for OP is mainly to prevent fractures, and reducing bone resorption or stimulating bone formation in this process are common means (Gennari et al., 2020). Based on this, the main therapeutic drugs for OP are anti-absorption drugs and anabolic agents (Li et al., 2021). Among them, bisphosphonates are the most widely used anti-bone resorption drugs, which can reduce bone turnover markers to a lower concentration before menopause and have achieved considerable results in reducing the fracture rate and the treatment of OP (Eastell and Szulc, 2017). However, the study revealed that the patients who were treated with bisphosphonates for 3 years or more were at an increased risk for osteonecrosis of the jaw (ONJ) and atypical femoral fractures (AFFs), but the absolute risks were low (Ayers et al., 2023). Moreover, poor adherence to bisphosphonate therapy is a major limiting factor in OP treatment, which is largely associated with gastrointestinal adverse events (Pagnotti et al., 2019). In addition, treatment using bisphosphonates can also cause other side effects, such as fever and myalgia, especially after the treatment is started (Ayers et al., 2023). It is worth noting that these side reactions caused by bisphosphonates can be alleviated by TCM, which is due to the characteristics of TCM with multiple approaches, multiple targets, and the four diagnostic methods of TCM, namely, observation, listening, asking, and cutting (Zhang et al., 2016).

Compared with the single treatment using bisphosphonates, TCM can develop personalized treatment plans according to the different physiques of patients. Specifically, for patients with poor spleen and stomach function, accompanied by general weakness, drugs with properties of strengthening the spleen and replenishing qi can be used; for patients with spleen and stomach disorders, accompanied by diarrhea and abdominal pain, drugs with an effect of strengthening the spleen and stopping diarrhea can be used; TCM can also improve blood circulation, promote the delivery of bone nutrients, and alleviate pain in patients with OP through methods such as acupuncture and moxibustion (Zhuo et al., 2022). Therefore, TCM has unique advantages in the treatment of OP, including alleviating symptoms, improving prognosis, and reducing fracture incidence.

However, the mechanism and pharmacodynamic components of TCM on OP treatment are still unclear, and the main limiting factors of its development are also problems that need to be solved urgently.

## 4 TCM in the treatment of OP based on syndrome differentiation

**FIGURE 2:**
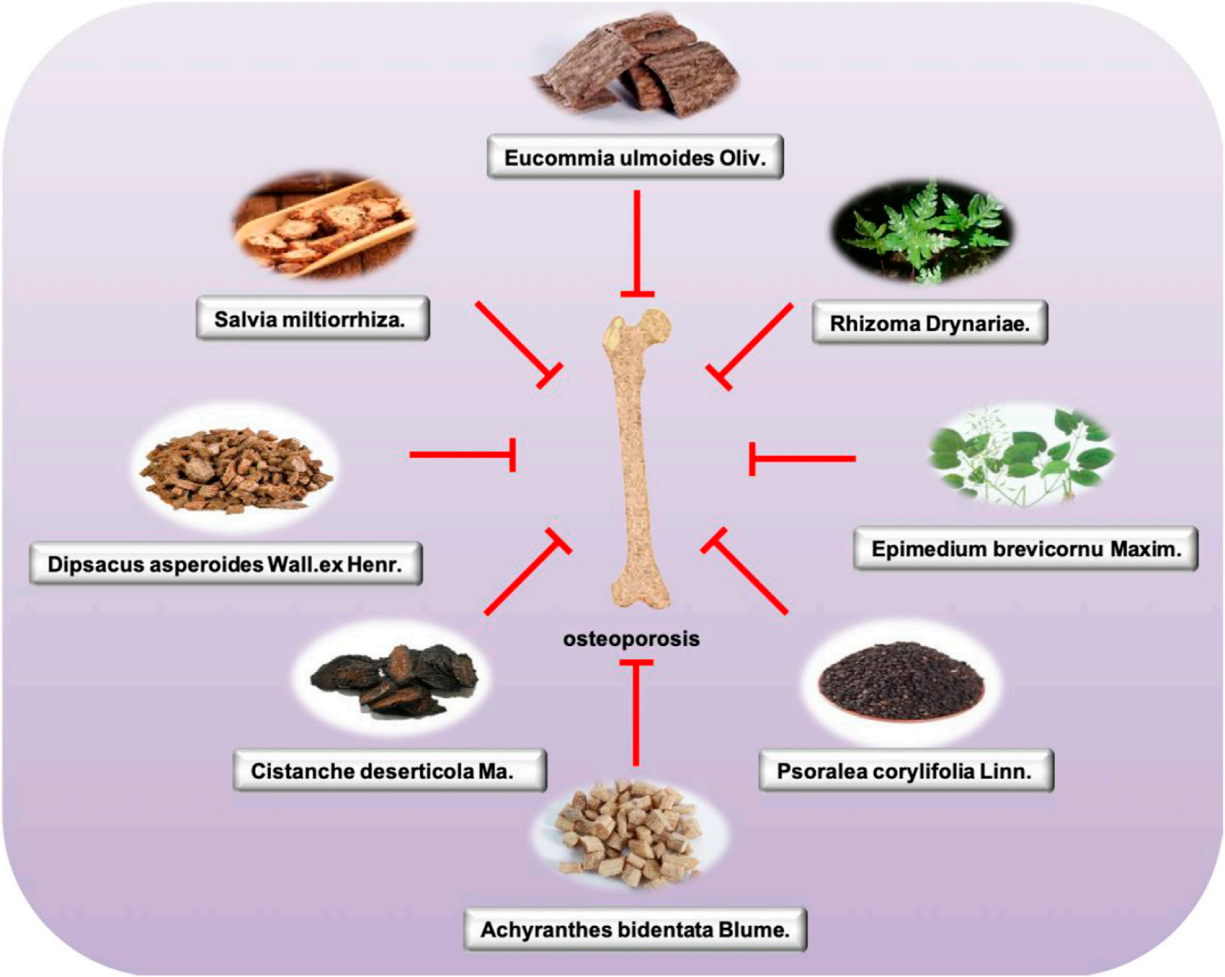
Main single herbs for treating osteoporosis.

TCM has a long history of being used to prevent and treat osteoporosis. According to the pathology and clinical manifestations of OP in modern medicine, OP in TCM can be classified into the scope of “ostealgia (Gu bi)” and “atrophic debility of bones (Gu wei)” according to “Nei jing.” Among them, kidney deficiency, blood stasis, and qi and yin deficiency are the main pathogenesis, and the disease location is bone. The nature is “deficiency of kidney essence, spleen deficiency, and nourishment loss,” whose features are blood stasis block (Mohammad et al., 2018). For the treatment of this disease, some TCM preparations, acupoint application, acupuncture, massage, and other TCM therapies are also applied to improve BMD. According to the TCM theory, bone diseases are closely related to the health status of the kidney. Therefore, people with kidney qi deficiency and kidney yin deficiency are more prone to suffer from OP. Traditional treatments mainly use the method of tonifying the kidney and strengthening the bone based on syndrome differentiation. In addition to drug treatment, acupuncture and acupoint application can effectively prevent and treat the disease. The research of single Chinese medicine and effective components mainly involves Epimedii Folium, Eucommiae Cortex, and Salviae Miltiorrhizae Radix et Rhizoma (Figure 2).

### 4.1 Herbal extracts

Chinese herbal medicine formulations with the advantages of multiple components and targets have been widely considered by researchers in the treatment of chronic disease (Cui et al., 2010). Bellavia et al. revealed the potential effects of flavonoids in bone resorption and promoting bone formation via a review of literature records in the last 5 years (Bellavia et al., 2021). In addition, saponins, iridoid glycosides, and lignans in Chinese herbal medicine also showed certain therapeutic effects on OP, whose mechanism was associated with Wnt/β-catenin, BMP/Smad, and mitogen-activated protein kinase (MAPK) pathways, RANKL/OPG signaling, and others (Zhang et al., 2016). For example, the dry leaf of *Epimedium brevicornu* Maxim. has a long history in the treatment of bone diseases in China due to its effect of tonifying kidney yang and strengthening muscles and bones. According to modern research, total flavonoids of *Epimedium brevicornu* Maxim. can treat osteoporotic distal radius fractures (Xue et al., 2016). Icariin (ICA), as the main active flavonoid glycoside in *Epimedium brevicornu* Maxim., has the effect of enhancing osteogenic activity through the regulation of the JNK/c-Jun signaling pathway, Wnt/β-catenin pathway, and Notch signaling pathway (Huang et al., 2020; Xu et al., 2019; Yu et al., 2020). In addition, Eucommiae Folium extract can promote the osteogenic differentiation of bone marrow mesenchymal stem cells (BMSCs) by regulating the Wnt/β-catenin signaling pathway and decreasing RANKL-induced bone resorption-related genes, such as TRAP (Cheng et al., 2019). The aqueous extract of Eucommiae Folium after salt roasting can promote the proliferation and differentiation of MC3T3-E1 cells, elevate the OPG level, and inhibit the secretion and expression of RANKL protein by regulating ERK, NF-κB, AKT pathways (Guan et al., 2021). Furthermore, Salviae Miltiorrhizae Radix et Rhizoma, also known as Danshen in Chinese, has a long history of being used to treat bone disorders (Guo et al., 2014). With the development of modern analytical techniques, many compounds have been isolated and identified from Danshen. Tanshinol is one of these compounds, and importantly, it can also play a role in reducing bone formation disorders through KLF15/PPARγ2 signaling (Yang Y.-j. et al., 2018). In addition, tanshinones are thought to inhibit osteoclast differentiation and may be a candidate for the treatment of OP (Lee et al., 2005). Moreover, Danshen has been reported to combine with Puerariae Lobatae Radix to alleviate OP through autophagy and oxidative stress-mediated osteoclast differentiation (Qin et al., 2021). Drynariae Rhizoma is introduced to improve glucocorticoid-induced OP by regulating the activity of osteoblasts and osteoclasts. Naringin has been identified as an effective anti-OP component of Drynariae Rhizoma (Peng C.-H. et al., 2022). Like naringin, the other five flavonoids, namely, aglycones, kurarinone, kushennol F, xanthogalenol, and sophoraflavanone G, have a potential protective effect against ovariectomized-induced osteoporosis, which may be related to the activation of endoplasmic reticulum signaling pathways (Wang et al., 2011). Achyranthis Bidentatae Radix is a TCM used to treat OP (Zhang M. et al., 2018). Polysaccharides have been the most studied in all components of Achyranthis Bidentatae Radix, and it has been reported that these polysaccharides can promote bone formation and, thus, play a potential role in anti-OP therapy (Zhang S. et al., 2018). In addition, Dipsaci Radix is a typical Chinese medicine used to treat OP by regulating immune-related pathways. In this study, ursolic acid and beta-sitosterol were shown to be the effective compounds (Zhang W. et al., 2019). Sweroside is the major active iridoid glycoside isolated from Dipsaci Radix. It has been reported that sweroside can exert beneficial effects on anti-OP by interacting with the membrane estrogen receptor-α and GPR30 to activate the p38 signaling pathway (Wu et al., 2020). As a triterpenoid saponin, asperosaponin VI exhibits anti-osteoclastogenic activity by inhibiting RANKL-induced osteoclast differentiation and function (Liu et al., 2019). Some compounds in Psoraleae Fructus display anti-OP activity by activating the ER-Wnt-β-catenin signaling pathway, among which isoflavones have the strongest activity (Cai et al., 2021). As a representative flavonoid, corylifol A can reduce ROS production through the activation of Nrf2, leading to the inhibition of osteoclast production and activation (Li et al., 2024). Similarly, corylin is also a flavonoid in Psoraleae Fructus, which increases the expression of osteogenic markers such as Runt-related transcription factor 2 (Runx2), osterix, type I collagen (Col1), and alkaline phosphatase (ALP), thus targeting the treatment of osteoblast-mediated OP (Yu TX. Y. et al., 2020). Cistanches Herba is an edible Chinese medicine that inhibits the RANKL/Rank-induced activation of downstream NF-κB and PI3K/AKT pathways and blocks the activity of the key osteoclastogenic proteins NFAT2 and c-Fos (Zhang B. et al., 2019). Cistanches Herba polysaccharide reduces RANKL-mediated ROS production in osteoclasts, which impairs osteoclastogenesis and bone resorption (Song et al., 2018). Cistanoside A is a phenylethanol glycoside compound isolated from Cistanches Herba, which has the potential to treat OP by downregulating TRAF6 (Xu et al., 2017). In summary, numerous TCM formulations and compounds derived from them have showed potential therapeutic effects in the prevention and treatment of OP (Table 1).

**TABLE 1:** Single herbs in osteoporosis.

Single herbs	Anti-osteoporotic compounds	Mechanism	Pathway	References
*Epimedium brevicornu* Maxim	Icariin, epimedin A, epimedin B, epimedin C, and icariside Ⅱ	Enhance osteogenic activity	EphB4/Ephrin-B2	Huang et al. (2020)
		Inhibit apoptosis in human MSCs	JNK/c-Jun	Cheng et al. (2019); Yu et al. (2020b)
		Promote the proliferation and differentiation of MSCs into osteoblasts	Wnt/β-catenin	Chen et al. (2016); Cheng et al. (2019)
			Notch	Cheng et al. (2019); Xu et al. (2019)
*Eucommia ulmoides* Oliv	5-(Hydroxymethyl)-2-furaldehyde	Promote the osteogenic differentiation of BMSCs	Wnt/β-catenin	Cheng et al. (2019)
		Promote the proliferation and differentiation of MC3T3-E1 and the ratio of OPG/RANKL	ERK, p38MAPK, NF-κB, and Akt	Guan et al. (2021)
*Salvia miltiorrhiza* Bge	Tanshinone VI, tanshinone ⅡA, salvianolic acid A, salvianolic acid B, and tanshinol	Block the expression of TRAF6 and NFTAc1 and develop osteoclast differentiation	NF-κB	Park et al. (2017)
		Activate AKT1, IL-6, VEGF-A, and mapk1 proteins and PI3K/Akt, IL-17, HIF-1, and AGE-RAGE pathways	PI3K/Akt, IL-17, HIF-1, and AGE-RAGE	Lee et al. (2020)
		and regulate the osteogenic differentiation function	Wnt/FOXO3a	Qin et al. (2020)
*Drynaria fortunei* (Kunze) J. Sm	Naringin, kurarinone, kushennol F, xanthogalenol, and sophoraflavanone G	Promote bone formation effectively and reduce bone resorption and related gene expression	PI3K-AKT, Wnt, and Es signaling pathways	Gan et al. (2019)
		Promote osteogenesis	JAK2/STAT3	Wang et al. (2021)
		Increase the expression of phosphorylated proteins	PI3K/AKT/mTOR	Ge and Zhou (2021)
		Treat osteoporotic fracture through angiogenesis	VEGF/VEGFR-2	Song et al. (2017)
*Achyranthes bidentata* Bl	Achyranthoside E, chikusetsusaponin Ⅳa, momordin Ⅰb, ecdysterone, daucosterol, quercetin, achyranthoside C dimethyl ester, achyranthoside C butyl dimethyl ester, achyranthoside E dimethyl ester, achyranthoside, and E butyl methyl ester	Enhance the expression of OB-related genes and differentiation of OBs	ERK signaling pathway	Hua and Zhang (2019)
*Dipsacus asper* Wall. ex Henry	Asperosaponin Ⅵ, ursolic acid, beta-sitosterol, and sweroside	Promote VEGF, angiogenesis, and the ratio of OPG/RANKL	RANKL/RANK/OPG/VEGF and PI3K/Akt/eNOS	Sun et al. (2019)
*Psoralea corylifolia* L.	Corylifol A and corylin	Induce OB differentiation and mineralization and enhance osteogenesis and mitochondria function	Es and Wnt/β-catenin signaling pathways	Yu et al. (2020a)
		Inhibit adipocyteformation and differentiation	Es and Akt/GSK-3β/β-catenin	Cao et al. (2019)
*Cistanche deserticola* Y. C. Ma	Cistanoside A and echinacoside	Inhibit the differentiation of osteoclast and the corresponding bone resorption	RANKL/RANK/TRAF6	Zhang et al. (2019b)
		Promote bone formation and prevent bone resorption	NF-κB and stimulation of PI3K/Akt	Xu et al. (2017)
		Attenuate the expression of OCs related genes and hydroxyapatite to suppress NFAT and MAPK activation	RANKL	Song et al. (2018b)
*Cornus officinalis* Sieb. et Zucc	Gallic acid, morroniside, loganin, sweroside, flavonol kaempferol, and cornuside I	Regulate the homeostasis of osteogenesis and osteoclast	PI3K-AKT and Wnt/β-catenin	Tang et al. (2023)
*Angelica sinensis* (Oliv.) Diels	Ferulic acid, ligustilide, and guaiacol	Promote osteoblast differentiation via the regulation of EGFR	GPR30/EGFR pathway	Yang et al. (2019)
*Coptis chinensis* Franch	Berberine, copisine, worenine, jatrorrhizine, and columbamine	Promote the proliferation and differentiation of osteoblasts as well as inhibit the production of osteoclasts to promote bone regeneration	Runx2	Zhang et al. (2021b)
*Cuscuta chinensis* Lam	Quercetin, kaempferol hyperoside, hyperin, p-hydroxycinnamic acid, and astragalin	Alleviate the increase of bone resorption markers and the decline of osteogenic markers	RANKL/OPG	Mo et al. (2019)
*Lycium barbarum* L.	Rutin	Alleviate age-related bone loss	BMPRIA/BMPRII/Noggin	Sun et al. (2023)
*Rehmannia glutinosa* Libosch	Catalpol and acteoside	Prevent bone loss and enhance osteoblastic bone formation	IGF-1/PI3K/mTOR	Gong et al. (2019)
*Polygonum multiflorum* Thunb	Emodin, polydatin, and 2,3,5,4′-tetrahydroxystilbene-2-O-*β*-D-glucoside	Ameliorate osteoporosis	MAPK	Lin et al. (2021)
*Curculigo orchioides* Gaertn	Curculigoside	Stimulate the osteogenic differentiation of MC3T3-E1 cells	BMP and Wnt	Wang et al. (2023)
*Phellodendron chinense* Schneid	Berberine	Promote osteoblast differentiation	p38 MAPK	Lee et al. (2008)

### 4.2 Traditional Chinese medicine formula

“Shen nong ben cao jing” recorded that medicine should be in harmony with the king and minister (Jun–chen–zuo–shi). Due to this theory, TCM preparations consisting of multiple herbs have attracted increasing international attention because of their characteristics and curative effects. Among these, OP Liuwei Dihuang pill (LWD), which mainly consists of *Rehmannia glutinosa* Libosch, *Paeonia suffruticosa* Andr, *Dioscorea opposita* Thunb*.*, Poria cocos (Schw.) Wolf, Alisma orientale (Sam.) Juz., and Cornus officinalis Sieb. et Zucc., was used in the treatment of OP, showing that miR-574 plays critical roles in osteoporosis, and kaempferol and quercetin actives may be the ingredients of LWD targeting MAPK1 to mediate MiR-574, thereby regulating the bone microenvironment and improving OP (Liu et al., 2022). Additionally, Shuai Bo et al. found that Qing’e Pill (QEP), consisting of *Eucommia ulmoides* Oliv., *Psoralea corylifolia* L., *Juglans regia* L., and *Allium sativum* L, could improve the microstructure of cancellous bone in ovariectomized mice by increasing the expression of β-catenin. Further study revealed that QEP could prevent osteoblast ferroptosis and increase osteogenesis (Shuai et al., 2019; Hao et al., 2022). Yangyang et al. confirmed that Yishen Bugu Ye (YSBGY) showed potential anti-osteoporotic effects through the modulation of the osteoblast/osteoclast balance and serum concentrations of inflammatory factors (Li et al., 2020). As a famous Chinese medicine preparation, Erzhi Wan (EZW) has a favorable anti-OP potential, mainly through inhibiting osteoclast bone absorption (Zhang et al., 2008). Zuogui Pill (ZGP) is a classic kidney-tonifying drug that can promote the osteogenic differentiation of bone marrow mesenchymal stem cells, which provides a scientific basis for its effective treatment of OP (Yang et al., 2018a). In addition, details of other traditional Chinese medicine formulas used in OP therapy are shown in Table 2.

**TABLE 2:** Prescription in osteoporosis.

Prescription name	Ingredients	Mechanism	References
Liuwei Dihuang pill (LWD)	Rehmannia glutinosa Libosch, Paeonia suffruticosa Andr, Dioscorea opposita Thunb., Poria cocos (Schw.) Wolf, Alisma orientale (Sam.) Juz., and Cornus officinalis Sieb. et Zucc	Improve the bone microenvironment, hormone, and enzyme activities	Liu et al. (2022)
Qing’e Pill (QEP)	Eucommia ulmoides Oliv., Psoralea corylifolia L., Juglans regia L., and Allium sativum L	Increase β-catenin expression	Shuai et al. (2019)
Yishen Bugu Ye (YSBGY)	Rhizoma Drynariae, Radix Polygoni Multiflori, Poria, Radix Dipsaci, Radix Paeoniae Alba, Radix Angelica sinensis, Radix Codonopsis, Radix Rehmanniae Preparata, Rhizoma Polygonati, Fructus Lycii, Pyritum, and Pericarpium Citri Reticulatae	Relate to regulate the OB/OC balance and inflammatory factors	Li et al. (2020)
Erzhi Wan (EZW)	Ligustrum lucidum Ait. and Eclipta prostrata (L.) L	Restraint of osteoclastic bone resorption	Zhang et al. (2008)
Zuogui pill (ZGP)	Rehmannia glutinosa Libosch, Dioscorea opposite Thunb, Lyciumbarbarum L., Cornus officinalis Sieb. et Zucc., Cyathula officinalis Kuan, Cuscuta chinensis Lam., *Cervus elaphus* Linnaeus, and Chinemys reevesii (Gray)	Promote the differentiation of osteoblasts and osteogenesis-related genes and reduce the adipocyte transcription	Yang et al. (2018b)
Xianlingubao Prescription (XLGB)	Epimedii Folium, Anemarrhenae Rhizoma, Salviae Miltiorrhizae Radix et Rhizoma, Psoraleae Fructus, Dipsaci Radix, and Rehmanniae Radix	IL-17, HIF-1, insulin resistance, Th-17 signaling pathway; promote blood circulation	Zhu and Hou (2020)
Hachimi-jio-gan (HJG)	*Rehmanniae radix, Corni fructus, Dioscoreae rhizome, Alismatis rhizome, Hoelen, Moutan cortex, Cinnamoni cortex, Aconiti tuber*	Produce qi and increase bone mass	An et al. (2016), Qu et al. (2020)
Erxian decoction (EXD)	Curculigo orchioides Gaertn., Epimedium brevicornu Maxim., Angelica sinensis (Oliv.) Diels, Morinda officinalis F.C. How, Phellodendron chinense C.K. Schneid, and Anemarrhena asphodeloides Bunge	Reduce TNF-α, osteoblast apoptosis, and purge Huo	Yang et al. (2021a)
Bu Zong Yi Qi Tang	Astragalus membranaceus (Fisch.) Bunge, Atractylodes macrocephala, Citrus reticulata Blanto, Cimicifuga foetida L., Radix Bupleuri, Panax ginseng C. A. Mey., Glycyrrhiza uralensis Fisch., and Angelica sinensis	Enhance BMD and elevate estrogen level in serum	Sakamoto et al. (2000)
Dang Gui Bu Xue Tang (DBT)	Astragalus membranaceus (Fisch.) Bunge var. mongholicus (Bunge) P. K. Hsiao, Angelica sinensis (Oliv.) Diels	Elevate BMD, MDA, and bone trabecula degradation and increase endogenous SOD activity	Xie et al. (2012)
Ba Wei Di Huang Wan	(Rhizome), Poria cocos (Schw.) Wolf (Sclerotium, Alisma orientale (Sam.) Juz., Cornus officinalis Sieb. et Zucc, Aconitum carmichaeli Debx., and Cinnamomum cassia Presl	Increase trabecular bone volume and BMD and improve the microstructure of the bone	Chen et al. (2012b)
Gu Ling Pian (GLP)	Drynaria fortunei (Kunze ex Mett.) J. Sm and Cuscuta chinensis Lam. *Cervus elaphus* Linnaeus	Increase MG-63 cells and regulate the ratio of OPG/RANKL via the p38 MARK pathway	Zhao et al. (2007)
Bu Shen Ning Xin Decoction (BSNXD)	Rehmannia glutinosa Libosch, Curculigo orchioides Gaertn., Cullen corylifolium (Linnaeus) Medikus, Hominis Placenta, Dioscorea opposita Thunb. Paeonia suffruticosa Andr., Atractylodes macrocephala Koidz., and Lycium barbarum L	Enhance osteoblastic proliferation and inhibit the apoptosis of osteoblasts through the MARK pathway activated by pERK	Wang et al. (2009)
Wu Jia Bu Gu recipe	Acanthopanax senticosus (Rupr. et Maxim.) Harms, Rehmannia glutinosa Libosch Achyranthes bidentata Bl., Astragalus membranaceus (Fisch.) Bunge var. mongholicus (Bunge)P. K. Hsiao, Angelica sinensis (Oliv.) Diels, and Ostrea gigas Thunberg	Increased ALP, serum Ca, and P, deposition of external calcium, production of collagen I, BMD maximum load, and elastic load, TBV%, TFS%, AFS%, and MAR	Fu et al. (2010)

## 5 Therapeutic mechanism of TCM on anti-osteoporosis

Although TCM has certain advantages in the treatment of osteoporosis, its therapeutic mechanism has not been fully elucidated, which is undoubtedly a huge challenge for researchers. In this study, we summarize and generalize the signaling pathways involved in OP, which may provide a certain theoretical basis for further elucidation of OP treatment with TCM. According to previous studies, Wnt/β-catenin, BMP-SMAD, MAPK, and RANK/NF-κB/OPG play a key role in OP (Wang et al., 2022; Wei et al., 2022) (Table3).

**TABLE 3:** Signaling pathways involved in osteoporosis.

Signaling pathways	Regulator	Mechanism	References
Wnt/β-catenin signaling pathway	Runx2	Directly regulate the expression of Runx2, thereby promoting the transdifferentiation of vascular smooth muscle cells and calcification of osteoblast	Cai et al. (2016)
	FoxO3	Runx2 could cooperate with FoxO3	Yuan et al. (2022)
	miR-29a	A downstream factor of Wnt/β-catenin signal transduction, could ameliorate age-induced osteoblast loss and osteoporosis	Lian et al. (2021)
	TGFβ	Induce the secretion of Wnt1, thereby combining bone resorption with bone formation	(Weivoda et al., 2016)
	IGF-1	Antagonized the Wnt/β-catenin signaling pathway by catalyzing the transcription of Axin2 and stabilizing the Axin1 protein	E. (2018), Lindsey and Mohan (2016), Zhang et al. (2019)
	Glucocorticoid	Can stimulate the differentiation of osteoblasts, thereby inhibiting bone formation through Wnt/β-catenin, BMPs, and other classical pathways	Compston (2018)
TGF-β/BMP signaling pathway	Smad2/3	Could be activated by TGF-β and then regulated the TGF-β-induced differentiation of chondrocyte and osteoblast in the Smad-dependent pathway	Kang et al. (2005)
	MAPKs	BMP signals could transduce the signal to the MAPK or Smad signaling pathway, which further regulated the transcription of related genes that are involved in the differentiation of osteoblasts and formation of bone	Wu et al. (2016)
	Others	BMP-2 can promote osteogenesis by regulating the expression of Runx2, ALP, and integrin-binding sialoprotein and activate osteoclast through the upregulation of TNF-α and NF-κB ligands	Ingwersen et al. (2022)
MAPK signaling pathway	ERK1/2	The MAPK pathway was related to osteoclastogenesis as well as bone resorption, and its mechanism may be associated with the phosphorylation of ERK1/2	Long et al. (2022)
	RUNX2	The expression of MAPK/p38MAPK could be increased by RUNX2, along with the increases of ALP, OCN, and OSX	Ren et al. (2022)
	AGEs	P38, ERK, and JNK can be activated by AGEs and contribute to the release of TNF-α, IL-1β, and IL-6	Wang et al. (2020)
RANKL/NF-κB/OPG signaling pathway	LGR4	Could competitively bind RANKL to RANK and block classical RANK signaling	Luo et al. (2016)
	TNF	Could regulate the expressions of FoxO1, Sod2, and catalase and accumulation of ROS, which were involved in the activation of the NF-κB pathway	Liao et al. (2016)
	Glucocorticoids	Contribute to the formation of osteoclast and expressions of RANKL and macrophage colony-stimulating factor (MCSF), inhibit the level of serum OPG, and activate and mature osteoclasts	Chotiyarnwong and McCloskey (2020)
	Estrogen	Could promote the accumulation of superoxide involved in bone remodeling and promote osteogenic differentiation	Karim et al. (2021)
PI3K/AKT signaling pathway	Alp, cbfa1, Col1a1, and OCN	The osteoblast-related gene expressions can be upregulated by the phosphorylation levels of PI3K and Akt	Xie et al. (2022)
mTOR signaling pathway	Glucocorticoids	Can promote the apoptosis and the autophagy of OB through inhibiting the mammalian target of rapamycin	Chotiyarnwong and McCloskey (2020)
ERα-AMPK-Sirt1 signaling pathway	LKBl	ER, including ER-α and ER-β, can directly increase the activity of LKBl, which is the most important upstream protein kinase of AMPK; the mutual promotion of AMPK and Sirt1 can also modulate the autophagy or apoptosis of osteoblast	Xiao et al. (2022)
JAK-STAT signaling pathway	IGF-I	Growth hormone facilitated longitudinal bone growth primarily via the production of hepatic IGF-I, and growth hormone receptor was activated and then induced the phosphorylation of the JAK-STAT signaling pathway	Lindsey and Mohan (2016)

### 5.1 Wnt/β-catenin signaling pathway

Wnt signaling is a critical signal transduction pathway and mainly regulates embryonic development and tissue regeneration (Nusse and Clevers, 2017). As a kind of secretory glycoprotein, Wnt-regulating signaling was associated with multiple genes and various receptors, all of which could regulate canonical β-catenin-dependent and non-canonical β-catenin-independent pathways (Wang et al., 2019). Among them, the canonical Wnt signaling pathway could be regulated at many levels, including negative regulation. During this process, DKK1, a negative regulator, can bind to the LRP receptor, thereby suppressing the Wnt signaling pathway. When cells were not exposed to Wnt signaling, major signaling components, such as β-catenin and receptors, were in a closed state (Nusse, 2012). When it was at an active state, Wnt signaling could activate the intracellular protein DVL and inhibit the degradation activity of the β-catenin degradation complex formed by GSK-3β. In non-canonical β-catenin-independent signaling pathways, Wnt could induce cytoskeletal re-arrangement through the activation of GTPase, including Rho and Rac (van Amerongen et al., 2008).

**FIGURE 3:**
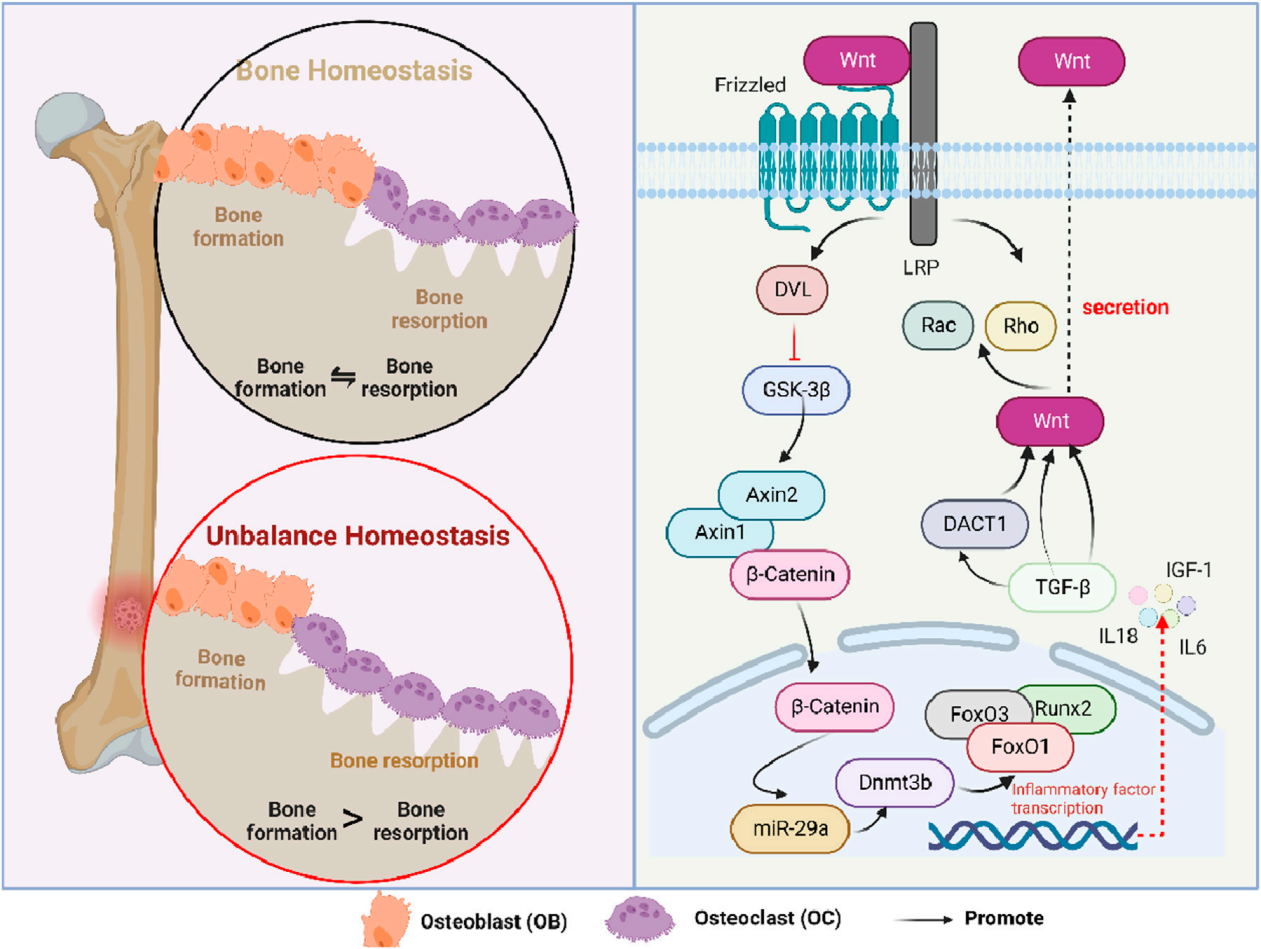
Role of the Wnt/β-catenin signaling pathway in osteoporosis. Interleukin, IL; insulin-like growth factor-1, IGF-1; lipoprotein receptor-related protein, LRP; disheveled, DVL; glycogen synthase kinase-3β, GSK-3β; DNA methyltransferase 3B, Dnmt3b; runt-related transcription factor 2, Runx2; forkhead box O, FoxO; dickkopf-1, DKK1; disheveled binding antagonist of beta-catenin 1, DACT1; and transforming growth factor-beta, TGF-β.

Wnt/β-catenin could regulate bone metabolism by controlling the differentiation and function of mesenchymal stem cells (Zhu et al., 2019), adipose-derived stem cells (Shao et al., 2017), osteoblasts, and osteoclasts. The specific mechanism of action was as follows: Runx2, a specific transcription factor, plays a vital role in osteoblast differentiation and chondrocyte maturation (Komori, 2018). Cai et al. found that Wnt/β-catenin could directly regulate the expression of Runx2, thereby promoting vascular smooth muscle cells to transdifferentiation (Cai et al., 2016). In addition, Runx2 could cooperate with the forkhead box protein O3 transcription factor (FoxO3) that belongs to a subclass of forkhead transcription factors (Yuan et al., 2022). Moreover, Runx2 could cross-conduce with the Wnt signal, all of which were involved in the elimination of superoxide, thereby remodeling normal bone. In addition, miR-29a, a downstream factor of Wnt/β-catenin, could ameliorate age-induced osteoblast loss and OP by targeting Dnmt3b-mediated FoxO3 methylation, upregulating the expressions of antioxidant proteins and DNA methylation (Lian et al., 2021). Similarly, FoxO1 was also involved in the degradation of spontaneous cartilage and the formation of osteoarthritis. Matsuzaki et al. found that the ectopic expression of FoxO1 could synergize with the stimulation of transforming growth factor-β (TGF-β), leading to the differentiation of macrophages along with the release of IL-1β (Matsuzaki et al., 2018). In addition, TGF-βin osteoclast can induce the secretion of Wnt1, thereby combining bone resorption with bone formation (Weivoda et al., 2016). Moreover, the paracrine secretion of DKK1 (a Wnt inhibitor) induced by TGF-β is essential for osteoclastogenesis. Subsequently, Esposito found that TGF-β could induce the biomolecular aggregation of DACT1, which suppresses the Wnt signaling pathway and promotes bone metastasis (Esposito et al., 2021). Furthermore, Zhang et al. found that insulin inhibited autophagy and promoted premature aging through the TGF-β pathway, thus inhibiting BMSC osteogenesis. (Zhang et al., 2020). IGF could directly affect the differentiation of osteoblasts and enhance the function of mature osteoblasts, thereby promoting the formation of bone collagen and bone matrix (Xian et al., 2012). During this process, IGF-1 antagonized the Wnt/β-catenin signaling pathway by catalyzing the transcription of Axin2 and stabilizing the Axin1 protein. Meanwhile, the IGF-1 receptor can phosphorylate and degrade β-catenin, activate GSK-3β, and degrade insulin receptor substrate 1, all of which contribute to glucose and bone metabolism (E., 2018; Lindsey and Mohan, 2016; Zhang B. et al., 2019) (Figure 3). Additionally, the physiological dose of glucocorticoid can stimulate the differentiation of osteoblasts, thereby inhibiting bone formation through BMPs and other classical pathways (Compston, 2018).

### 5.2 TGF-β/BMP signaling pathway

Similar to the role of the Wnt/β-catenin signaling pathway, the TGF-β/BMP signaling pathway also plays a crucial regulatory role in the body, especially in the homeostasis of postnatal bone and differentiation of mesenchymal cells into osteoblasts (Chen G. et al., 2012; Wu et al., 2016), which may be due to DNA synthesis and cell replication. Specifically, there are three main forms of TGF-β in mammals: TGF-β1, TGF-β2, and TGF-β3 (Derynck and Erine, 2019), all of which could be activated and bind to TGF-β I receptors (TβRI) and two type II receptors (TβRII). Subsequently, TGF-β could transmit its signals to the Smad signaling pathway both in canonical-dependent and non-canonical-independent forms (Wu et al., 2016). For example, Smad2/3 could be activated by TGF-β, regulating the TGF-β-induced differentiation of chondrocyte and osteoblast through the Smad pathway. Moreover, Smad2/3 also recruited HDACs 4/5 and inhibited Runx2 function, all of which could participate in osteoblast differentiation (Kang et al., 2005), while in the Smad-independent pathway, TGF-β accelerates the proliferation and differentiation of osteoblast by regulating MAPK and Smad2/3 signaling pathways (Matsunobu et al., 2009). In addition, MAPK could positively regulate the function of Runx2, contributing to the differentiation of MSCs (Li et al., 2009).

**FIGURE 4:**
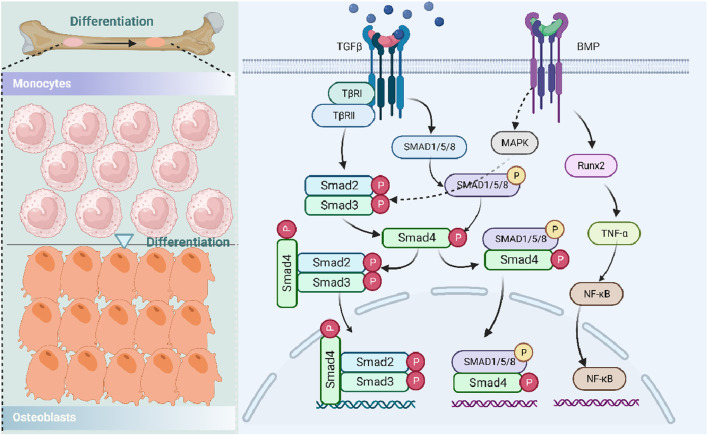
Role of the TGF-β/BMP signaling pathway in osteoporosis. Transforming growth factor-beta, TGF-β; TGF-β type I receptor, TβR; runt-related transcription factor 2, Runx2; histone deacetylases, HDACs; mitogen-activated protein kinase, MAPK; bone morphogenetic protein, BMP; tumor necrosis factor-α, TNF-α; and nuclear factor-κB, NF-κB.

In bone, BMP signals were also mediated by their receptors and formed complex bodies with them, further regulating the transcription of related genes involved in the differentiation of osteoblast and formation of bone (Wu et al., 2016). Among BMPs, BMP-2 could promote osteogenesis by regulating the expression of Runx2, ALP, and integrin-binding sialoprotein and activate osteoclast through the upregulation of TNF-α and NF-κB ligands (Ingwersen et al., 2022). Moreover, BMP/Smad1 could regulate the activity of atonal homolog 8 and inhibit the expression ratio of RANKL/OPG, thereby regulating the osteoclast number negatively and promoting bone resorption and loss (Yahiro et al., 2020) (Figure 4).

### 5.3 MAPK signaling pathway

MAPK is a class of conserved serine/threonine protein kinases and plays a role in cell proliferation, differentiation, and apoptosis (Zhang W, 2003). In mammals, three families of MAPK have been identified: JNK kinase, extracellular signal-regulated protein kinase (ERK), and p38 MAPK. Among these, the MAPK-mediated pathway is within an enzymatic cascade, which comprises at least three continuously activated enzymes (Widmann et al., 1999).

**FIGURE 5:**
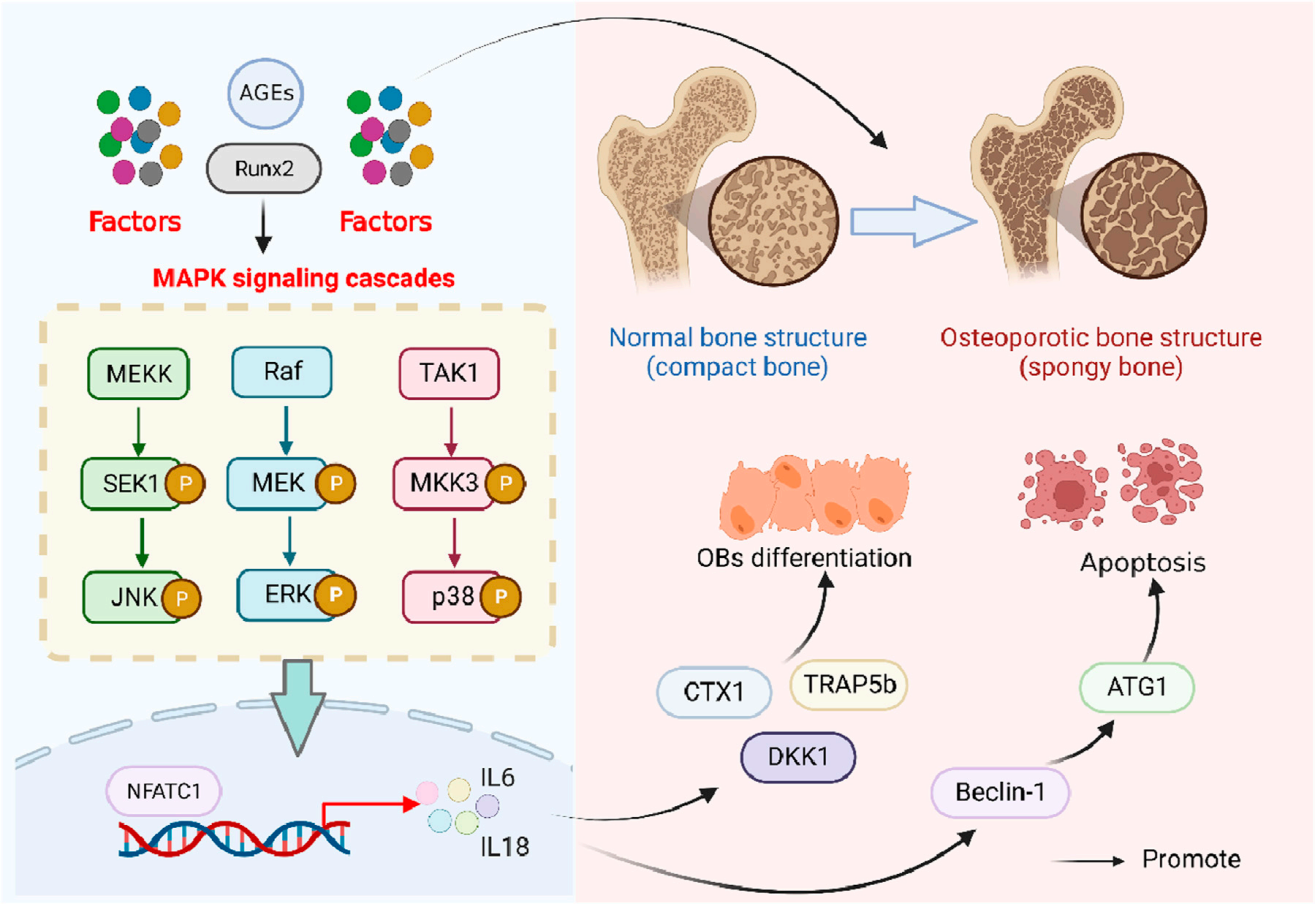
Role of the MAPK signaling pathway in osteoporosis. Advanced glycation end products, AGEs; runt-related transcription factor 2, Runx2; SAPK/Erk kinase 1, SEK1; Jun N-terminal kinase, JNK; mitogen-activated protein kinase kinase, MEK; mitogen-activated protein kinase kinase kinase, MEKK1; extracellular signal-regulated kinases, ERK; transforming growth factor-β-activated kinase 1, TAK1; MAP kinase kinase-3, MKK3; C-terminal telopeptide of type I collagen, CTX1; tartrate-resistant acid phosphatase isoform 5b, TRAP5b; dickkopf-1, Dkk1; anti-human t-lymphocyte globulin 1, ATG1; nuclear factor of activated T-cells 1, NFATC1; and interleukin, IL.

In OP, the MAPK pathway was related to the osteoclastogenesis as well as bone resorption, which was due to its role in the phosphorylation of ERK1/2 that could, in sequence, regulate the transcription and expression of the main osteoclast transcription factor, such as a recombinant nuclear factor of activated T-cells, cytoplasmic 1 (NFATc1) (Long et al., 2022). In addition, Ren et al. found that the expression of MAPK/p38MAPK could be increased by RUNX2, along with the increases of ALP, OCN, and OSX, all of which were associated with the osteoblast differentiation and downregulation of autophagy genes, including Beclin-1, ATG1, and p62 (Ren et al., 2022). In addition, the MAPK signaling pathway also takes part in high-glucose-caused osteoclast differentiation, which was associated with Dickkopf-1 and tartrate-resistant acid phosphatase 5B (TRAP5b), c-terminal telopeptides of type 1 (CTX1), cathepsin K, and Nqo1 (Ren et al., 2022). Moreover, downstream of MAPK, pathways such as P38, ERK, and JNK can be activated by AGEs and contribute to the release of TNF-α, IL-1β, and IL-6 (Wang et al., 2020) (Figure 5).

### 5.4 RANKL/NF-κB/OPG signaling pathway

**FIGURE 6:**
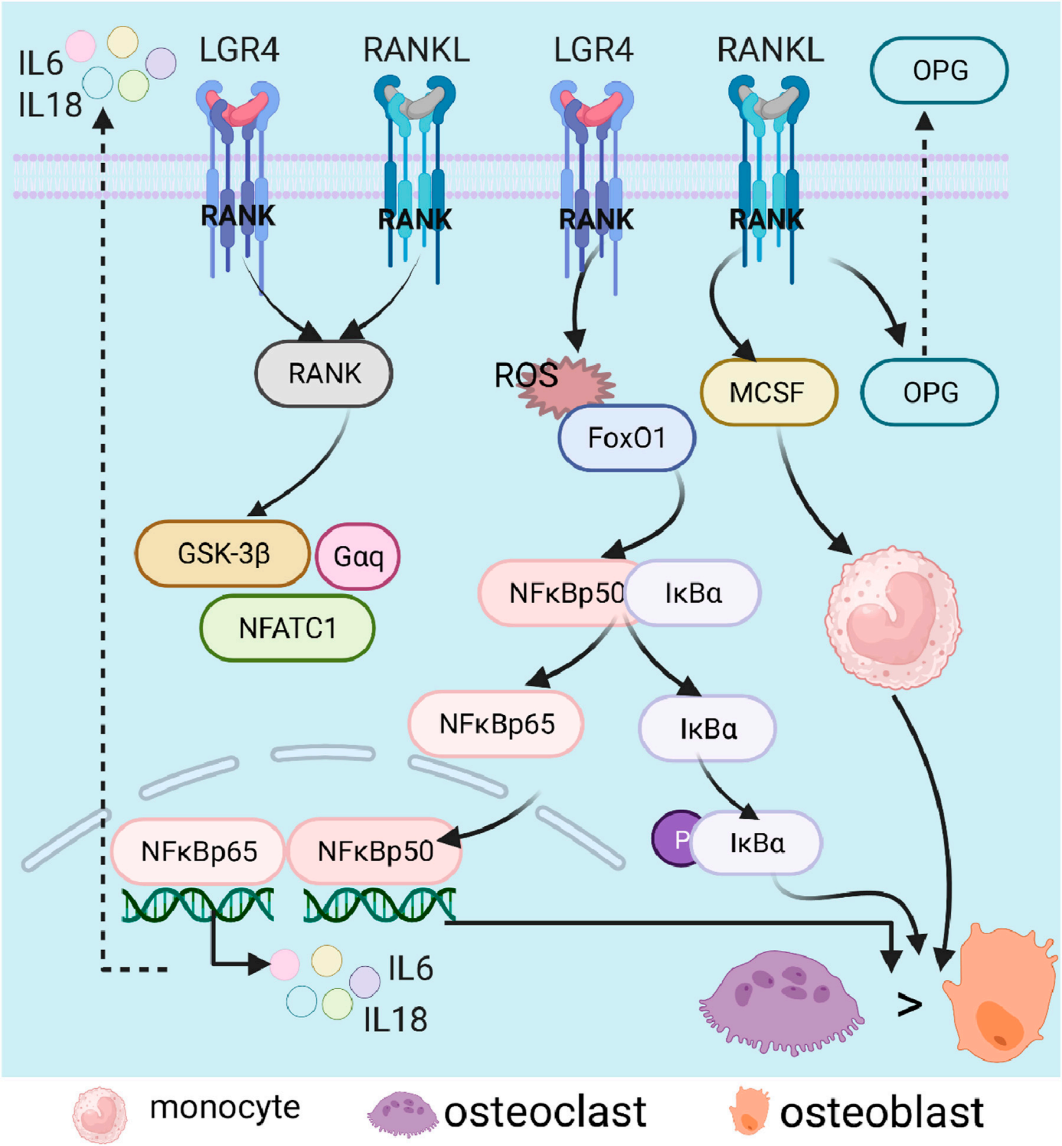
Role of the RANK/NF-κB (RANKL)/OPG signaling pathway in osteoporosis. Nuclear factor-κB, NF-κB; nuclear factor kappa B, RANK; nuclear factor-κB ligand, RANKL; inhibitor of NF-κB α, IκBα; osteoprotegerin, OPG; interleukin, IL; glycogen synthase kinase-3β, GSK-3β; nuclear factor of activated T-cells 1, NFATC1; forkhead box O, FoxO; reactive oxygen species, ROS; and macrophage colony-stimulating factor, MCSF.

The RANKL/NF-kB/OPG pathway, mainly involved in osteoclast formation (Whyte, 2006), is a process initiated by the binding of RANKL to RANK (Yang W. et al., 2021). RNAKL, known as TNF superfamily member 11 (TNFSF11), mainly regulates the differentiation of osteoclasts and the formation of OP (Luo et al., 2016). During osteoclast differentiation, leucine-rich repeat-containing G-protein-coupled receptor 4 (LGR4) (a receptor of RANKL) could competitively bind RANKL to RANK and block classical RANK way, along with activations of the glycogen synthase kinase-3 (GSK3)-β, α subunit of inhibitory G protein (Gαq), and NFATC1 (Luo et al., 2016). In addition, TNF could regulate the activation of the NF-κB pathway through the expressions of FoxO1, Sod2, and catalase and accumulation of ROS (Liao et al., 2016); under normal conditions, NF-κB can form a dimer with IκBα in the cytoplasm; upon stimulation, the dimer was dissociated, along with NF-κB entering the nucleus and IκBα phosphorylation, resulting in the synthesis and release of inflammatory factors, all of which promoted osteoclast formation, osteoclast differentiation, and osteolysis (Zou et al., 2020). Additionally, long-term use or overdose of glucocorticoids contribute to the formation of osteoclast and expressions of RANKL and macrophage colony-stimulating factor (MCSF), inhibiting the level of serum OPG and activating osteoclasts (Chotiyarnwong and McCloskey, 2020). Moreover, estrogen could promote the accumulation of superoxide involved in bone remodeling and promote osteogenic differentiation. In postmenopausal osteoporosis, deficiency of estrogen led to unbalanced bone homeostasis, a decrease in OPG expression, and increases in RANKL and M-CSF (Karim et al., 2021) (Figure 6).

### 5.5 Other pathways related to the pathogenesis of osteoporosis

In addition to the above signal paths, relevant research showed that PI3K/AKT/mTOR, ERα-AMPK, GH/IGF (Lindsey and Mohan, 2016), and calcium signaling pathways also participate in OP pathogenesis. The osteoblast-related gene expressions, including Alp, cbfa1, Col1a1, and osteocalcin (OCN), can be upregulated by the phosphorylation of PI3K and Akt (Xie et al., 2022). Low levels of glucocorticoids can promote the apoptosis and autophagy of OB through inhibiting the mammalian target of rapamycin (mTOR) pathway (Chotiyarnwong and McCloskey, 2020). ER, including ER-α and ER-β, can directly increase the activity of LKBl, which is the most important upstream protein kinase of AMPK. In addition, the mutual promotion of AMPK and Sirt1 can also modulate the autophagy or apoptosis of osteoblasts, suggesting that the ERα-AMPK-Sirt1 signaling pathway may play an important role in OP (Xiao et al., 2022). Furthermore, growth hormone facilitated longitudinal bone growth primarily via the production of hepatic IGF-I, and the growth hormone receptor was activated and then induced the phosphorylation of the Janus kinase (JAK)-signal transducers and activators of transcription (STAT) pathway (Lindsey and Mohan, 2016). Of note, the JAK-STAT pathway can disrupt normal bone remodeling by targeting osteoclasts and osteoblasts in the joint and in the joint exoskeleton (Damerau et al., 2020).

## 6 Clinical or preclinical studies of TCM in OP treatment

TCM has unique advantages in the treatment of osteoporosis, which mainly lies in the overall regulation of the balance of yin and yang of the human body (Hu et al., 2019), fundamentally improving bone metabolism and repair, and preventing and treating the occurrence and development of OP from various aspects (Qian et al., 2021). As we mentioned, TCM has made certain progress in the treatment of OP, which is not only reflected in preclinical research but has also achieved good results in clinical research. At present, the National Medical Products Administration has approved many Chinese patent drugs for the treatment of OP in China. Among them, the Xianling Gubao capsule (tablet) (Xiao et al., 2022), Gushukang capsule (granule) (Li et al., 2022), Jintiange capsule (Liang et al., 2022), and Qianggu capsule (Huang et al., 2022) are more widely used. In addition, the characteristic therapy of OP in Chinese medicine includes acupuncture, moxibustion, treatment by way of pasting on acupuncture points, massage, and so on (Peng Z. et al., 2022). To sum up, there are various methods for the treatment of OP by TCM, but it is worth noting that appropriate therapies should be selected according to different syndrome types under the guidance of the TCM theory.

## 7 Conclusion and future prospects

The pathogenic factors and related pathways in OP reported in this review provide a basis for better elucidating the pathogenesis of osteoporosis. TCM, including syndrome differentiation, single herbs, and prescription, shows a unique advantage in the treatment of osteoporosis, which undoubtedly points out the direction for researchers. However, besides the abovementioned diseases related to osteoporosis, whether there are other diseases that can affect OP or not needs to be studied.

Although TCM has certain advantages in the treatment of osteoporosis, the material basis is unclear, which brings challenges to clinical application. Compared with the single target of chemical drugs, the multi-pathway and multi-target characteristics of TCM are not only advantages but also disadvantages, which is also a major challenge for the internationalization of TCM. This review summarizes the potential of TCM in the treatment of osteoporosis, but its specific mechanism is worth further research. The solution to this problem will be beneficial to the long-term development of TCM in the treatment of osteoporosis.
